# Analysis of prodromal symptoms and need for short-term prophylaxis in angioedema patients under long-term prophylaxis

**DOI:** 10.1186/s13023-025-03562-1

**Published:** 2025-02-01

**Authors:** Robin Lochbaum, Thomas K. Hoffmann, Jens Greve, Janina Hahn

**Affiliations:** Department of Oto-Rhino-Laryngology, Head and Neck Surgery, Ulm University Medical Center, Frauensteige 12, Ulm, 89075 Germany

**Keywords:** Berotralstat, C1 inhibitor, Dental procedure, Lanadelumab, Surgery, Trigger

## Abstract

**Background:**

Patients with hereditary angioedema (HAE) experience recurrent, unpredictable episodes of edema. These swellings are often preceded by prodromal symptoms. HAE management includes acute treatment, long-term prophylaxis (LTP), and short-term prophylaxis (STP) before procedures with a risk of swelling. The effects of LTP on prodromal symptoms and the necessity for STP in patients on LTP remain unclear.

**Methods:**

A questionnaire-based study involving HAE and AAE patients receiving LTP was conducted. Changes in prodromal symptoms and the incidence of procedures with an increased risk of swelling, including surgeries, dental procedures, and endoscopies were assessed.

**Results:**

A total of 26 patients were included in the study. Among them, 18 experienced zero to three attacks since starting LTP. Abdominal attacks constituted 60% of all attacks, followed by swellings of the extremities and head and neck. The most frequently reported trigger factors were stress and mechanical stimuli, followed by infections. 9 patients reported surgical procedures, with 8 using STP. Of these, 4 experienced breakthrough attacks, including one laryngeal attack. 105 dental procedures were reported, with STP used for only one. Only one angioedema attack occurred after an intervention without STP. For endoscopies, 7 procedures were reported, 3 of which were performed under STP. Two abdominal attacks were reported by the same patient, both without prior STP. Prodromal symptoms remained consistent in type but varied in intensity and frequency under LTP.

**Conclusions:**

For dental procedures, the mandatory use of STP in HAE patients on effective LTP should be reconsidered, provided acute treatment is available and other trigger factors are absent.

**Supplementary Information:**

The online version contains supplementary material available at 10.1186/s13023-025-03562-1.

## Background

Hereditary Angioedema (HAE) is a rare genetic disorder characterized by recurrent episodes of severe swelling in various parts of the body, including the extremities, face, gastrointestinal tract, and airways [1]. The disease is typically caused by mutations in the gene that encodes C1 inhibitor (C1INH). Bradykinin is the most important mediator of edema in HAE [2]. The disease can be diagnosed genetically or more easily by blood testing showing reduced C4 complement levels and reduced C1INH concentration and/or C1INH function [3].

There is another type of HAE called HAE with normal C1INH (HAE-nC1INH). In these cases, patients have similar clinical characteristics but normal C1INH and C4 values [4]. Acquired C1INH deficiency (AAE-C1INH) is a condition that also resembles HAE but has a late onset (usually after 40 years of age) and reduced C1INH values due to consumption potentially caused by oncologic or autoimmune disorders [5].

Patients with HAE or AAE have different and individual clinical courses, ranging from no attacks at all to several attacks per week. Angioedema in HAE can be very painful and life-threatening when edema of the upper airways occurs. Some factors known to trigger HAE attacks include physical trauma (such as minor injuries, dental work, or surgery, especially with intubation), emotional stress (both positive and negative), hormonal changes, and infections [6].

Many patients with HAE report prodromal symptoms, which can serve as early warning signs before an HAE attack occurs. Various individual symptoms can occur, with the most reported ones being erythema marginatum (a non-itchy, annular rash), fatigue, tingling sensations, nausea, abdominal pain, and mood changes [7].

According to the most recent international guidelines, the management of HAE includes acute treatment, short-term prophylaxis (STP), and long-term prophylaxis (LTP) [8]. It should be noted that the guidelines and approval studies were not designed for HAE-nC1INH patients, but these patients are usually treated in the same way as no other options are known or approved. This also applies to patients with AAE-C1INH when no underlying disease can be detected (and therefore treated) or when “watchful waiting” is the recommended approach [9].

For optimal treatment response, every attack should be treated as early as possible to optimize the effectiveness of the acute medication. For acute treatment, C1INH concentrates (plasma-derived or recombinant) can be administered. Another first-line option is the bradykinin B2 receptor antagonist icatibant, a subcutaneously administered drug [8].

To achieve complete control of the disease by reducing the frequency and burden of attacks, LTP can be started in HAE patients. Currently, the following drugs are first-line therapeutic options for LTP: C1INH concentrates (IV or SC), the monoclonal antibody lanadelumab that inhibits plasma kallikrein and is administered subcutaneously, and berotralstat, the only oral treatment option in HAE, which is also a kallikrein inhibitor [8].

STP is also known as situational prophylaxis or pre-interventional prophylaxis. For first-line STP, plasma-derived C1INH concentrates can be administered intravenously [10]. The current international guideline states, “We recommend considering STP before medical, surgical, or dental procedures as well as exposure to other angioedema attack-inducing events.” [8].

One open topic in the current international HAE guideline, which the present study aimed to address, is the following: “It is possible that STP should be handled differently in patients with a complete response to effective LTP, such as subcutaneous C1INH, lanadelumab, or berotralstat. No recommendation on this can be given at this time, as data are lacking. We encourage studies and reports on the need for STP in patients on LTP.” [8].

Additionally, prodromal symptoms in HAE are known to individually forecast an HAE attack. It is yet unknown if and how the type, severity and frequency of prodromal symptoms are influenced in HAE patients under LTP. This was the second question addressed in the present analysis.

## Methods

A questionnaire was developed with a focus on the two mentioned open topics (supplementary material; in German and English). Prodromal symptoms and their changes under LTP were examined. Procedures with an increased risk of swelling, particularly operations, dental procedures, and endoscopies, were investigated. Further questions addressed the general course of HAE/AAE and the efficacy of LTP.

The questionnaire was sent to all angioedema patients under LTP who met the following inclusion criteria at the angioedema center of the authors. One inclusion criterion was treatment with LTP for at least six months. Further inclusion criteria were that the patients had one of the following diagnoses: (a) laboratory-confirmed HAE due to C1INH deficiency (HAE-C1INH) with accordingly reduced C4 values, as well as reduced C1INH activity and/or C1INH concentration, or (b) HAE-nC1INH with a genetically proven mutation of one of the genes recognized to cause HAE-nC1INH, or (c) laboratory-confirmed AAE-C1INH. Patients with HAE-nC1INH but without a known mutation were excluded. In all cases, C1INH IV was administered for STP according to the current guideline recommendations.

Statistical analysis was performed with GraphPad Prism. McNemar’s test was used to investigate whether individual prodromal symptoms changed significantly under LTP. Four groups were formed: (1) Patients who did not suffer from prodromal symptoms before starting LTP but then developed prodromal symptoms, (2) patients without prodromal symptoms who did not experience any prodromal symptoms during the further course, (3) patients who reported prodromal symptoms before LTP and still continued to suffer from them under LTP and (4) patients who reported prodromal symptoms before LTP but who no longer suffered from them during the further course under LTP.

The study protocol was approved by the local ethics department (Nr.: 326/23), and written informed consent was obtained from all patients.

## Results

### Patients’ characteristics and treatment

26 patients who had been on LTP for at least six months completed the questionnaire. Of these, 16 (61.5%) were female and 10 (38.5%) were male. The mean age at the time of the survey was 43 years (range: 14 to 73 years). The mean age at the first onset of angioedema symptoms was 12 years (range: 4 to 33 years). Regarding the first onset of symptoms, one patient with AAE-C1INH was analysed separately (first onset of symptoms: 62 years). The mean number of years from the first onset of HAE symptoms to diagnosis was 8 years (range: 0 to 31 years). Among the included patients, 23 had HAE-C1INH, 1 had AAE-C1INH, and 2 were diagnosed with HAE-nC1INH.

The patients had been on LTP for a median duration of 5 years (range: 1 to 24 years). All patients used C1INH IV or icatibant SC for the acute treatment of attacks. Table 1 summarizes the reported trigger factors for attacks. Multiple answers were possible.

**Table 1:**
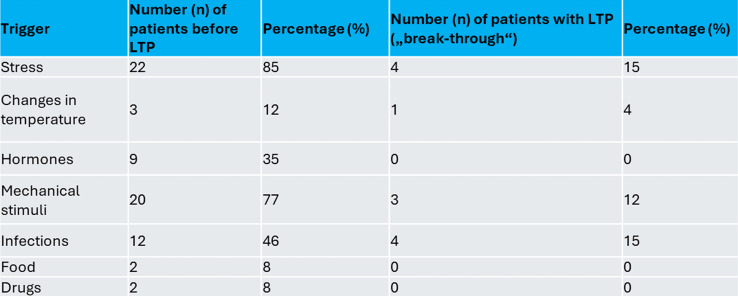
The reported trigger factors for angioedema attacks before and under long-term prophylaxis (LTP), multiple answers were possible for one patient

20 patients had lanadelumab SC, four berotralstat PO, one C1INH SC and one C1INH IV. Ten out of 26 patients switched their LTP drug during their course of disease, most often switching from C1INH SC to lanadelumab SC due to the less frequent interval of application. The frequency of attacks was the most frequently mentioned reason for initiating LTP (92%), followed by pain due to swelling (65%), concern because of unforeseen attacks (62%) and restrictions in everyday life (54%), with several answers possible for one patient.

### Clinical course under LTP

**Fig. 1:**
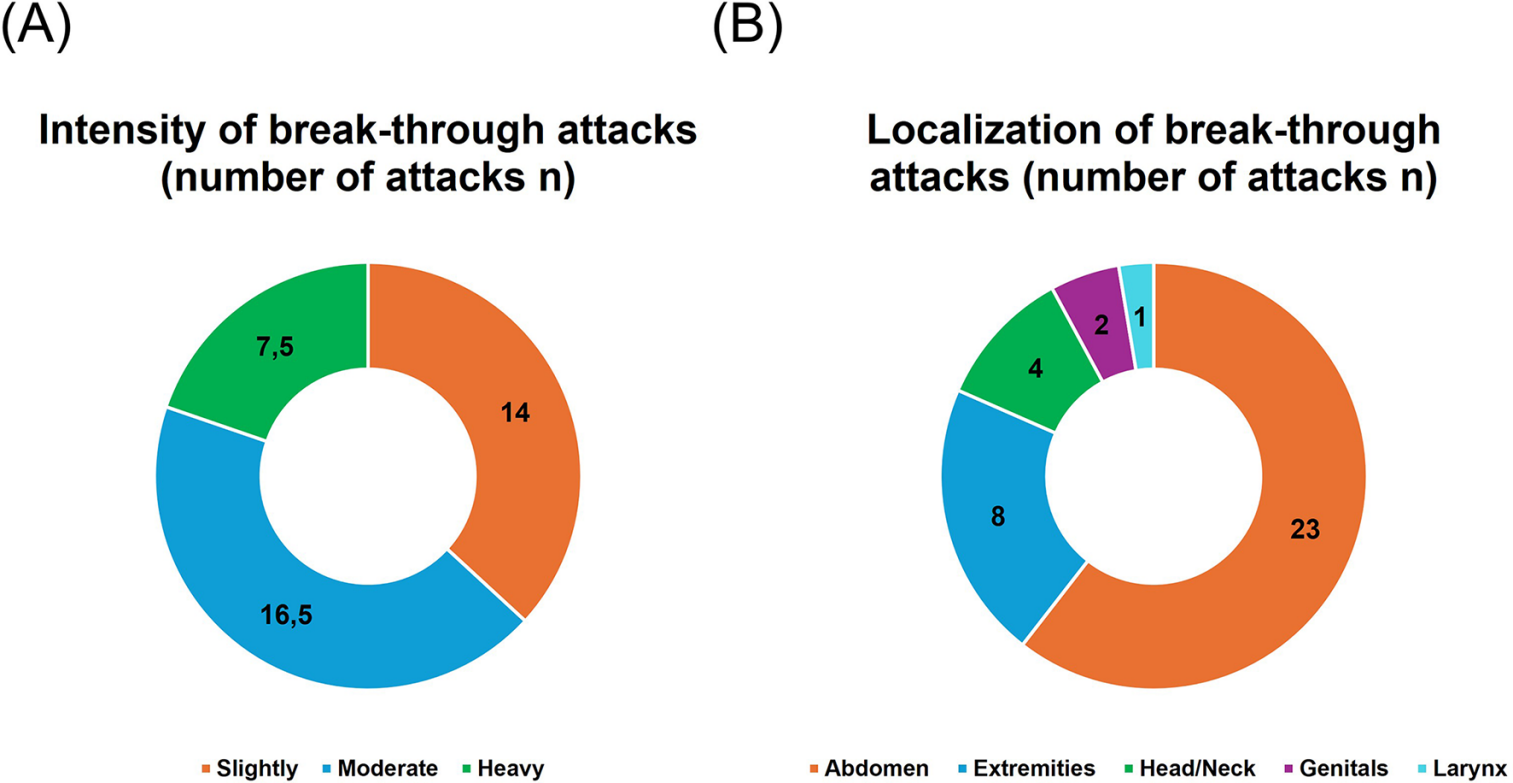
The intensity (**A**) and localization (**B**) of break-through attacks; number of attacks n; 16 patients

10 out of 26 patients did not experience any angioedema attacks since starting LTP. 8 reported 1 to 3 attacks since LTP was started, 2 reported 4 to 10 attacks, and 3 reported more than 10 attacks. The intensity and localization of the breakthrough attacks are summarized in Fig. 1a and b. Trigger factors for the breakthrough attacks were most often stress and infections (Table 1). Hormones were not mentioned as a trigger factor for breakthrough attacks under LTP by any of the 26 patients.

### Interventions

**Fig. 2:**
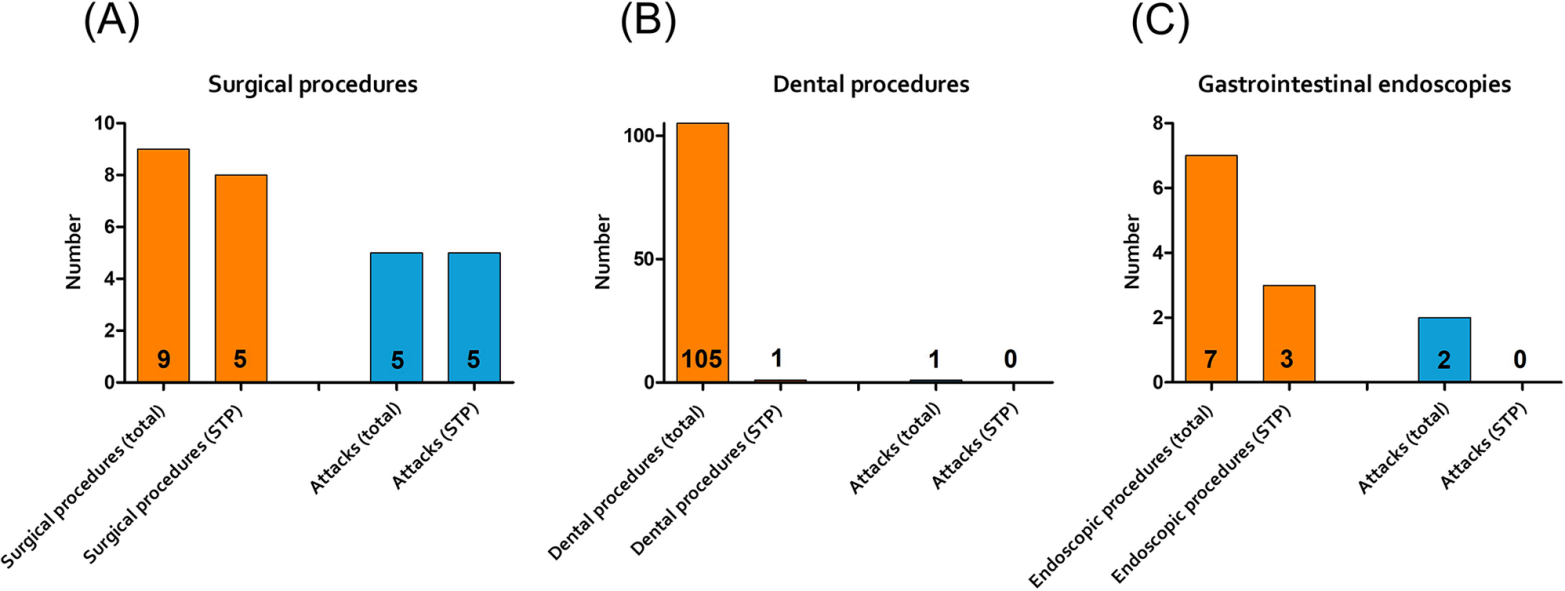
The results of the surgical treatments (**A**), dental procedures (**B**) and gastrointestinal endoscopies (**C**) considering the number of attacks with and without short-term prophylaxis (STP)

Since the initiation of LTP, 9 surgical procedures were reported by 8 patients (summarized in Table 2). All interventions except the caesarean sections required intubation anesthesia. The surgical procedures included two caesarean sections, one surgical treatment of a fractured leg, one arthroscopy, one thyroidectomy, one surgical procedure of the uterus (myoma), one total endoprothesis of the hip, one vasectomy, and one nephrectomy. In five cases, an angioedema attack occurred after the surgical treatment. Figure 2a shows the results of the surgical treatment considering the number of attacks with and without STP. All but one attack was localized in the region of the surgical intervention; in one patient, the edema was laryngeal leading to prolonged intubation and ventilation.

**Table 2:**
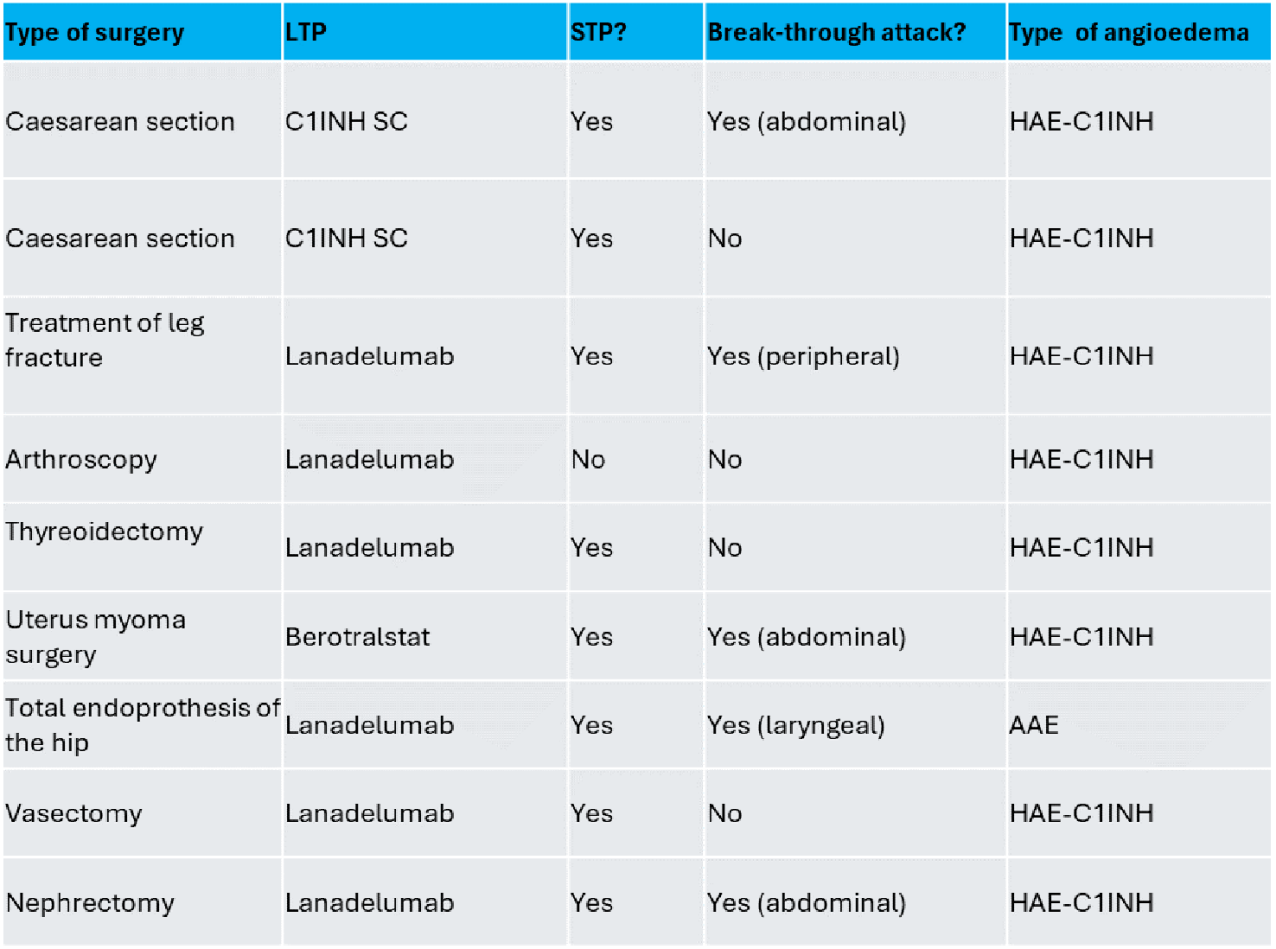
Summary of all surgical treatments under long-term prophylaxis (LTP). For short-term prophylaxis (STP), C1INH 1ooo IE IV were used (HAE-C1INH = hereditary angioedema due to C1INH deficiency; AAE = acquired angioedema)

Regarding dental procedures, 105 procedures were reported by 19 patients. Figure 2b shows the results; in summary, only one angioedema attack occurred after an intervention without STP, even though only one patient took STP before one of the procedures.

Since the initiation of LTP, 7 endoscopies (gastroscopy and/or colonoscopy) were performed in 5 patients (Fig. 2c). Three endoscopies were performed under STP; 2 abdominal attacks were reported by the same patient, both without prior STP.

There was no significant correlation between the number of attacks with or without STP and the number of breakthrough attacks since the initiation of LTP using Spearman´s rank correlation analysis.

### Prodromal symptoms

**Fig. 3:**
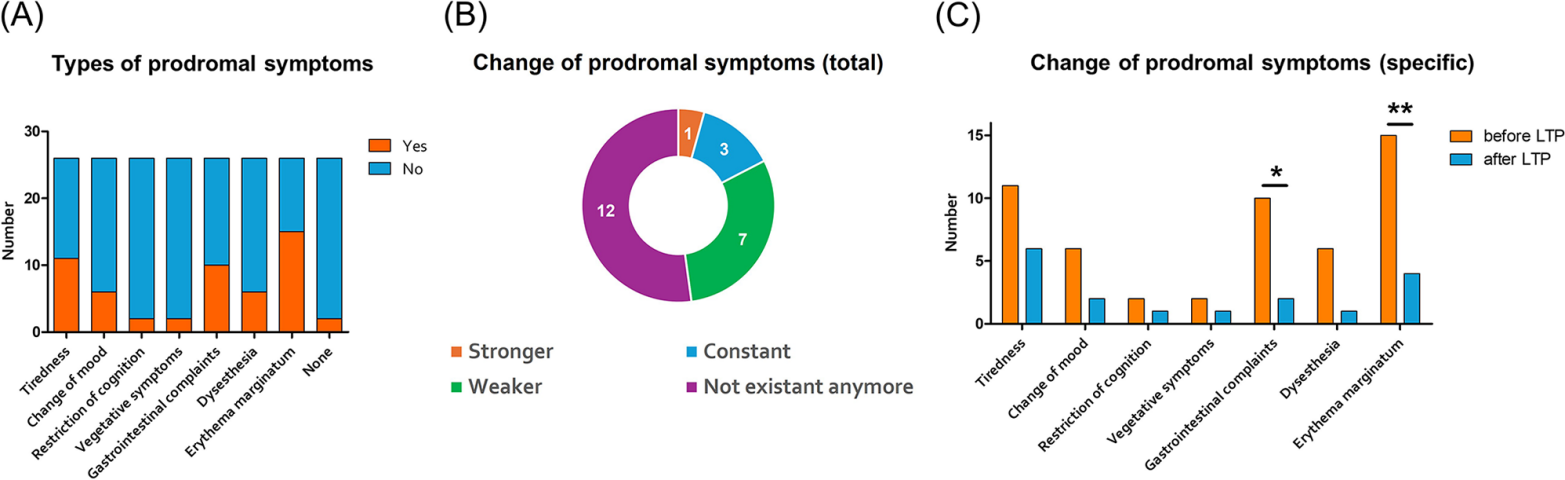
The types of prodromal symptoms (**A**), the change of prodromal symptoms in total under long-term prophylaxis (LTP; **B**) and an analysis of all reported types of prodromal symptoms under LTP (**C**). *n* = 26, * *p* = 0.0133, ** *p* = 0.0026; McNemar’s test

The types of prodromal symptoms are summarized in Fig. 3a. Two patients had never experienced prodromal symptoms and were excluded from the analysis of changes in prodromal symptoms under LTP. Of the remaining 24 patients, 12 did not report any prodromal symptoms under LTP. Seven patients reported a decreased frequency of prodromal symptoms under LTP. In 4 patients, the frequency did not change, and in 1 patient, one prodromal symptom– tiredness - increased under LTP (Fig. 3b).

Figure 3c shows an analysis of all reported types of prodromal symptoms under LTP. There was a consistent reduction in the occurrence of each prodromal symptom type, which was significant for gastrointestinal complaints (*p* = 0.0133) and erythema marginatum (*p* = 0.0026). 45% of patients with persistently recurrent prodromal symptoms went on to have breakthrough attacks after the occurrence of the prodromi. At the same time, the number of patients who administered on-demand medication after the occurrence of prodromal symptoms decreased.

## Discussion

The present results are among the first published findings on the topic of STP under LTP and prodromal symptoms under LTP in patients with bradykinin-mediated angioedema. The authors are aware of two other studies by colleagues from Berlin, Germany and Milan, Italy on the topic of STP under LTP with lanadelumab [10, 11]. The study from Milan involved only dental prodecures. No publications are currently available on the evaluation of prodromal symptoms under LTP. Both topics directly affect the daily management recommendations for patients with HAE or AAE.

One point for discussion is the chosen methodology. Ideally, a prospective, randomized evaluation would be advantageous but is hardly feasible for ethical reasons. The number of included patients must be considered in the context of the low incidence of this rare disease - it is estimated that 1,500 to 2,000 patients in Germany are diagnosed with HAE. One of the two studies to date on STP under LTP is the real-life evaluation of patients on LTP with lanadelumab by Buttgereit and colleagues from Charité Berlin, which evaluated data from 22 patients for this question [11]. A retrospective Italian study published by Zanichelli and colleagues analyzed 20 HAE and AAE patients with a total of 75 dental and oral procedures: 27 procedures were preceded by STP only, 13 were performed in patients regularly taking LTP without STP, 33 procedures were under both LTP and STP, and two procedures took place without either STP or LTP. Two attacks were reported: one in an HAE patient without any treatment and one in an AAE-C1INH patient without LTP but with prior STP [10]. In the present study, it was also the AAE-C1INH-patient who suffered from the only laryngeal attack after surgery with intubation, although STP was given. Since it is known that in AAE-C1INH patients the catabolism of C1INH is faster, these patients seem to have an increased risk of developing post-interventional edema and should therefore take STP (which is off-label in AAE) and be supervised afterwards accordingly.

As already mentioned, one patient with AAE-C1INH and two patients with HAE-nC1INH with confirmed mutations were also included in the present analysis. This inclusion can be debated, as the clinical courses of AAE-C1INH and HAE-C1INH, as well as HAE-nC1INH and HAE-C1INH, show clear differences. Additionally, the therapy for AAE-C1INH and HAE-nC1INH is off-label. However, since all patients and disease patterns showed a good response (defined as positive results in AECT, AE-QoL and reduced frequency of HAE attacks) to LTP and breakthrough attacks can occur under therapy in all three disease patterns, it was decided to include these patients in the evaluation. This approach is consistent with the Berlin evaluation by Buttgereit et al. and the Milan evaluation by Zanichelli et al. [11].

The topic of a new definition of prodromal symptoms under LTP is a newly discussed issue at congresses on bradykinin-mediated angioedema. In principle, attacks in HAE patients should be treated as early as possible, as the pharmacokinetics of the medications result in a better therapeutic response with earlier treatment. In the present evaluation, the frequency and intensity of prodromal symptoms was in most of the patients significantly reduced under LTP, although the nature of the symptoms remained the same. Approximately half of the prodromal symptoms were followed by an HAE attack. A prospective study including 119 HAE attacks associated with prodromal symptoms (but without information about their current treatment) concluded that many patients experience a prodrome before at least one of their attacks, and 64% can predict an oncoming attack by having a prodrome [7]. Thus, the “warning function” of prodromal symptoms remains under LTP. Since no attack followed approximately half of the prodromal symptoms and the overall frequency was significantly reduced in our results, general recommendations for acute therapy cannot be given based solely on the occurrence of a prodromal symptom. However, the patients appear to be more relaxed about prodromal symptoms under LTP, as evidenced by the reduced use of on-demand medication.

In the following, the present results are compared with the evaluation from Berlin [11]: the spectrum of interventions, with a predominance of dental procedures, is comparable. In the Berlin study, only patients under lanadelumab treatment were evaluated, which also constituted most patients in the present evaluation. The approach and outcomes of dental procedures are similar in both studies. Each study showed that patients rarely used STP for over 100 dental interventions. Nevertheless, both the Berlin study and the present study reported only one swelling incident after dental procedures, corresponding to a rate of less than 1%. These results are also supported by the study from Milan, although it is not directly comparable due to the different forms of therapy (STP versus STP and LTP versus LTP alone versus none) [10].

Patients in the Berlin study were overall more restrictive in using STP: only two patients used situational prophylaxis, compared to the present evaluation where STP was used in 8 out of 9 surgical operations and 3 out of 9 endoscopies. Almost paradoxically, the results for breakthrough attacks differ. Despite not using STP for these interventions, there were no swellings in the evaluation by Buttgereit and colleagues, while in the present study, 5 out of 10 surgical interventions resulted in breakthrough attacks—all of which were under STP. Gastro- and colonoscopies did not lead to any angioedema attacks in the Berlin study, whereas in the present study, two mild attacks (both in the same patient) occurred during a total of 7 endoscopies, each without prior STP administration [11].

Stress is one of the most common triggers for an HAE attack. Since (dental) medical procedures typically mean stress for patients, this is an important consideration for healthcare providers managing HAE and AAE patients. Creating a stress-free environment by having acute medication ready and assuring the patient of the provider’s capability to handle any potential attack is crucial. Communication between HAE centers and local healthcare providers is essential for this.

In summary, the data from the present study, combined with the data from Berlin and Milan, do not support the necessity of mandatory STP under LTP in HAE patients. For dental procedures, the mandatory use of STP in HAE patients on effective LTP should be reconsidered, provided acute treatment is available and other trigger factors are absent. Breakthrough attacks were particularly rare during dental procedures and endoscopies, despite minimal STP use. The results of other surgical interventions should be interpreted with caution, given the overall small number of procedures per center. In addition to that, the situation seems to be different in AAE-C1INH patients. However, these results alone, contrary to initial expectations, show that many HAE patients remained attack-free without STP, and those who experienced swelling did so even under STP. It is not possible to interpret from the present data what impact LTP had, as it is unclear how the course would have been without LTP.

The discussion should include the goal of reducing STP. Fortunately, C1INH preparations have very few side effects, so one can argue that STP provides protective effects with minimal risk. Nevertheless, any unnecessary medication administration is a relief for the patient and simplifies pre-interventional management. Additionally, economic factors are relevant: HAE treatment medications are expensive and should be used judiciously. Reducing STP is another argument for LTP, which generally provides a significant quality of life improvement for severely affected patients.

However, the authors emphasize that the conclusion that reducing STP under effective LTP is possible should not endanger any HAE or AAE patient. For example, increased stress from not applying STP could lead to more breakthrough attacks. Treatment decisions should be made through shared decision-making, with the presence of effective and sufficient acute therapy as the foundation of any therapeutic approach.

## Electronic supplementary material

Below is the link to the electronic supplementary material.

Supplementary Material 1

Supplementary Material 2

## Data Availability

The authors confirm that most of the data supporting the findings of this study are available within the article and further data are available on request from the corresponding author.
