# Supplementary material for: Analysis of prodromal symptoms and need for short-term prophylaxis in angioedema patients under long-term prophylaxis
Source: Orphanet J Rare Dis. 2025 Feb 1;20:47. doi: 10.1186/s13023-025-03562-1 (PMC11786469; doi:10.1186/s13023-025-03562-1)
Supplement: Supplementary file 1 — Supplementary Material 1 [file 13023_2025_3562_MOESM1_ESM.pdf]

Universitätsklinikum Ulm – HNO-Klinik - 89070 Ulm

**Universitätsklinik und**
**Hochschulambulanz für**
**Hals-Nasen-Ohren-Heilkunde,**
**Kopf- und Hals-Chirurgie**
**Frauensteige 12, D-89075 Ulm**

**Ärztlicher Direktor**
**Prof. Dr. T. Hoffmann**
Frauensteige 12
D-89075 Ulm

**Sekretariat**
Marion Reith, Brigitta Clases
ent.department@uniklinik-ulm.de
T: 0731 500-59501 / 03
F: 0731 500-59502

**Sektion für**
**Phoniatrie/Pädaudiologie**
Leiterin: Frau Prof. Dr. A.-K. Rohlfs
T: 0731 500-59701
F: 0731 500-59702

# Fragebogen für Patienten mit hereditärem Angioödem (HAE) unter Langzeitprophylaxe

Thema: Eine Fragebogen-gestützte Analyse von Prodromalsymptomen und der Notwendigkeit einer Kurzzeitprophylaxe bei Patienten mit hereditärem Angioödem unter Langzeitprophylaxe.

Geschlecht: Geburtsjahr:

1. Wann sind bei Ihnen die ersten Symptome des Angioödems aufgetreten?

   ..............................................................................................................................
   ..............................................................................................................................

2. Wann wurde die Diagnose „hereditäres Angioödem" bei Ihnen gestellt?

   ..............................................................................................................................
   ..............................................................................................................................

**UNIVERSITÄTSKLINIKUM ULM** Anstalt des Öffentlichen Rechts, Sitz Ulm | UST-ID. Nr. DE147040060
Vorsitzender des Aufsichtsrats: Clemens Benz. Vorstand: Prof. Dr. Udo X. Kaisers (Vorsitzender),
Bettina Rottke (Stellv.), Prof. Dr. Florian Gebhard, Prof. Dr. Thomas Wirth, Robert Jeske
Sparkasse Ulm IBAN: DE16 6305 0000 0000 1064 78 BIC: SOLADES1ULM

3. Seit wann nehmen Sie eine Langzeitprophylaxe ein? (Jahreszahl)

   ..........................................................................................................................

4. Welches Medikament nehmen Sie für die Langzeitprophylaxe ein?
   □ Lanadelumab (Takhzyro) alle 2 Wochen 300mg
   □ Lanadelumab (Takhzyro) alle 4 Wochen 300mg
   □ Lanadelumab (Takhzyro) alle ________ Wochen 300mg
   □ Berinert alle 2-3 Tage nach Gewicht dosiert
   □ Cinryze alle 2-3 Tage nach Gewicht dosiert
   □ Orladeyo 150 mg einmal täglich
   □ Sonstiges: .........................................

5. Was war die Hauptursache für den Einsatz der Langzeitprophylaxe? (Mehrfachnennungen möglich!)

   □ Häufige Schwellungen
   □ Sehr starke Schmerzen bei den Schwellungen
   □ Gefährliche Schwellungen (z.B. im Kehlkopf)
   □ Viele Fehltage bei der Arbeit/Schule
   □ Starke Einschränkung und Belastung durch das HAE im Alltag
   □ Sorge, dass Attacken unvorhergesehen auftreten könnten
   □ Sonstiges:...........................................................................................................
   ..........................................................................................................................

6. Welche Faktoren lösen bei Ihnen Schwellungen aus?

   □ Stress (positiver und negativer Art)
   □ Temperaturänderungen
   □ Hormonelle Faktoren (z.B. Menstruation)
   □ Mechanische Reize (z.B. Verletzungen)
   □ Infekte (Schnupfen, Erkrankungen)
   □ Sonstiges:...........................................................................................................
   ..........................................................................................................................

7. Hatten Sie Schwellungen / Bauchschmerzattacken seit Einnahme der Langzeitprophylaxe?
   ➔ Bitte aufzählen soweit möglich

| **Datum** | **Möglicher Auslöser**<br><br>**(z.B. Erkältung)** | **Ort der Schwellung**<br><br>**(z.B. Arm, Bauchkrämpfe)** | **Intensität der Schwellung (mild, moderat, schwer)** |
|---|---|---|---|
| | | | |
| | | | |
| | | | |
| | | | |
| | | | |
| | | | |
| | | | |
| | | | |
| | | | |

8. Welches Medikament nehmen Sie aktuell zur Akutbehandlung einer Durchbruchattacke?

   □ Icatibant 30 mg subkutan
   □ Berinert gewichtsadaptiert intravenös
   □ Sonstiges: ..........................................................................................................
   ..........................................................................................................................

9. Hatten Sie seit Einnahme der Langzeitprophylaxe eine Operation? Wenn ja: welche und wann? Falls mehrere durchgeführt wurden, diese bitte einzeln aufzählen.
   ..........................................................................................................................
   ..........................................................................................................................
   ..........................................................................................................................
   ..........................................................................................................................

10. Wenn Frage 8 mit **ja** beantwortet wurde: hatten Sie vor der Operation ein weiteres Medikament („Kurzzeitprophylaxe“) zusätzlich eingenommen? Wenn ja: welches? (z.B. Berinert i.v. nach Körpergewicht). Falls mehrere durchgeführt wurden, diese bitte einzeln aufzählen.
    ....................................................................................................................
    ....................................................................................................................
    ....................................................................................................................
    ....................................................................................................................

11. Wenn Frage 8 mit **ja** beantwortet wurde: trat nach der durchgeführten Operation eine Schwellung / Bauchkrämpfe auf? Wenn ja, wo war sie, wie stark war sie (mild, moderat oder schwer) und wurde diese behandelt? Falls mehrere durchgeführt wurden, diese bitte einzeln aufzählen.
    ....................................................................................................................
    ....................................................................................................................
    ....................................................................................................................
    ....................................................................................................................

12. Hatten Sie seit Einnahme der Langzeitprophylaxe einen zahnärztlichen „Eingriff“? Wenn ja was? (Bitte auch professionelle Zahnreinigung angeben). Falls mehrere durchgeführt wurden, diese bitte einzeln aufzählen.
    ....................................................................................................................
    ....................................................................................................................
    ....................................................................................................................
    ....................................................................................................................

13. Wenn Frage 11 mit **ja** beantwortet wurde: hatten Sie vor dem Eingriff ein weiteres Medikament („Kurzzeitprophylaxe“) zusätzlich eingenommen? Wenn ja: welches? (z.B. Berinert i.v. nach Körpergewicht). Falls mehrere durchgeführt wurden, diese bitte einzeln aufzählen.
    ....................................................................................................................
    ....................................................................................................................
    ....................................................................................................................
    ....................................................................................................................

14. Wenn Frage 11 mit **ja** beantwortet wurde: trat nach dem durchgeführten zahnärztlichen Eingriff eine Schwellung / Bauchkrämpfe auf? Wenn ja, wo war sie, wie stark war sie (mild, moderat oder schwer) und wurde diese behandelt? Falls mehrere durchgeführt wurden, diese bitte einzeln aufzählen.
    ........................................................................................................................
    ........................................................................................................................
    ........................................................................................................................
    ........................................................................................................................

15. Hatten Sie seit Einnahme der Langzeitprophylaxe eine Magen- oder Darmspiegelung? Wenn ja was? Falls mehrere durchgeführt wurden, diese bitte einzeln aufzählen.
    ........................................................................................................................
    ........................................................................................................................
    ........................................................................................................................
    ........................................................................................................................

16. Wenn Frage 15 mit **ja** beantwortet wurde: hatten Sie vor dem Eingriff ein weiteres Medikament („Kurzzeitprophylaxe") zusätzlich eingenommen? Wenn ja: welches? (z.B. Berinert i.v. nach Körpergewicht) Falls mehrere durchgeführt wurden, diese bitte einzeln aufzählen.
    ........................................................................................................................
    ........................................................................................................................
    ........................................................................................................................
    ........................................................................................................................

17. Wenn Frage 15 mit **ja** beantwortet wurde: trat nach der durchgeführten Spiegelung eine Schwellung / Bauchkrämpfe auf? Wenn ja, wo war sie, wie stark war sie (mild, moderat oder schwer) und wurde diese behandelt? Falls mehrere durchgeführt wurden, diese bitte einzeln aufzählen.
    ........................................................................................................................
    ........................................................................................................................
    ........................................................................................................................
    ........................................................................................................................

18. Kam es bei Ihnen seit Einnahme der Langzeitprophylaxe zu einem anderen Ereignis, das bei Ihnen Schwellungen auslöst? (z.B. Krankheit, Infektion, Reise etc.)? Wenn ja, bitte auflisten.
    ...........................................................................................................................
    ...........................................................................................................................
    ...........................................................................................................................
    ...........................................................................................................................

19. Bitte Charakterisieren Sie die Schwellungen nach den in Frage 18 gelisteten Ereignissen. Wo waren sie, wie stark waren sie (mild, moderat oder schwer) und wurden diese behandelt?
    ...........................................................................................................................
    ...........................................................................................................................
    ...........................................................................................................................
    ...........................................................................................................................

20. Gab es vor dem Beginn der Langzeitprophylaxe Symptome, welche einer Schwellungsattacke vorausgingen (z.B. ausgeprägte Müdigkeit, Stimmungsänderungen, Kribbeln, Übelkeit, etc.)? Wenn ja, listen Sie diese bitte auf:
    ...........................................................................................................................
    ...........................................................................................................................
    ...........................................................................................................................
    ...........................................................................................................................

21. Wie haben Sie reagiert, wenn diese Symptome aufgetreten sind (z.B. abgewartet, die Bedarfsmedikation gegeben, körperliche Schonung, etc.)?
    ...........................................................................................................................
    ...........................................................................................................................
    ...........................................................................................................................
    ...........................................................................................................................

22. Haben Sie nach Einleitung der Langzeitprophylaxe weiterhin diese Symptome? Wenn, ja, welche?
    ...........................................................................................................................
    ...........................................................................................................................
    ...........................................................................................................................
    ...........................................................................................................................

23. Folgten diesen Symptomen weiterhin Schwellungen/Bauchschmerzattacken? Wenn mehrere Symptome angegeben wurden, diese bitte einzeln listen:
    ..........................................................................................................................
    ..........................................................................................................................
    ..........................................................................................................................
    ..........................................................................................................................

24. Haben sich diese Symptome in ihrer Art, Häufigkeit, Intensität o.Ä. seit Durchführung der Langzeitprophylaxe geändert? Wenn ja, wie?
    ..........................................................................................................................
    ..........................................................................................................................
    ..........................................................................................................................
    ..........................................................................................................................

25. Wie reagieren Sie inzwischen unter der Langzeitprophylaxe, wenn diese Symptome auftreten (z.B. abwarten, die Bedarfsmedikation geben, körperliche Schonung, etc.)?
    ..........................................................................................................................
    ..........................................................................................................................
    ..........................................................................................................................
    ..........................................................................................................................

26. Hatten Sie allgemein schon einmal einen rötlichen Hautausschlag, ggf. vor einer Schwellung? („Erythema marginatum")
    ..........................................................................................................................
    ..........................................................................................................................
    ..........................................................................................................................
    ..........................................................................................................................

27. Hatten Sie seit Einnahme der Langzeitprophylaxe schon einmal einen rötlichen Hautausschlag, ggf. vor einer Schwellung? („Erythema marginatum")
    ..........................................................................................................................
    ..........................................................................................................................
    ..........................................................................................................................
    ..........................................................................................................................

28. Worin sehen Sie selbst den größten Vorteil der Langzeitprophylaxe?
.........................................................................................................................
.........................................................................................................................
.........................................................................................................................
.........................................................................................................................

29. Gibt es etwas, was Ihrer Meinung nach bei der Langzeitprophylaxe besser sein könnte?
.........................................................................................................................
.........................................................................................................................
.........................................................................................................................
.........................................................................................................................

Vielen Dank für das Beantworten der Fragen!

Ihr Angioödem-Team der HNO-Universitätsklinik Ulm
