# Supplementary material for: Analysis of prodromal symptoms and need for short-term prophylaxis in angioedema patients under long-term prophylaxis
Source: Orphanet J Rare Dis. 2025 Feb 1;20:47. doi: 10.1186/s13023-025-03562-1 (PMC11786469; doi:10.1186/s13023-025-03562-1)
Supplement: Supplementary file 2 — Supplementary Material 2 [file 13023_2025_3562_MOESM2_ESM.pdf]

universität ulm
DIE DEUTSCHEN UNIVERSITÄTSKLINIKA®
UNIVERSITÄTS KLINIKUM ulm

Universitätsklinikum Ulm – HNO-Klinik - 89070 Ulm

Universitätsklinik und Hochschulambulanz für Hals-Nasen-Ohren-Heilkunde, Kopf- und Hals-Chirurgie Frauensteige 12, D-89075 Ulm

Ärztlicher Direktor
Prof. Dr. T. Hoffmann
Frauensteige 12
D-89075 Ulm

Sekretariat
Marion Reith, Brigitta Clases
ent.department@uniklinik-ulm.de
T: 0731 500-59501 / 03
F: 0731 500-59502

Sektion für Phoniatrie/Pädaudiologie
Leiterin: Frau Prof. Dr. A.-K. Rohlfs
T: 0731 500-59701
F: 0731 500-59702

Questionnaire for Hereditary Angioedema (HAE)-patients under long-term prophylactic (LTP) treatment

A questionnaire-based analysis of prodromal symptoms and the need for short-term prophylaxis (STP) in patients with hereditary angioedema (HAE) on long-term prophylaxis LTP).

Gender: Year of birth:

1. When did the first symptoms of angioedema occur in your case?

....
....

2. When was the diagnosis of "hereditary angioedema" made in your case?

....
....

UNIVERSITÄTSKLINIKUM ULM Anstalt des Öffentlichen Rechts, Sitz Ulm | UST-ID. Nr. DE147040060
Vorsitzender des Aufsichtsrats: Clemens Benz. Vorstand: Prof. Dr. Udo X. Kaisers (Vorsitzender), Bettina Rottke (Stellv.), Prof. Dr. Florian Gebhard, Prof. Dr. Thomas Wirth, Robert Jeske
Sparkasse Ulm IBAN: DE16 6305 0000 0000 1064 78 BIC: SOLADES1ULM

3. Since when do you take LTP? (year)

   ....

4. Which medication do you take for LTP?
   □ Lanadelumab (Takhzyro) every 2 weeks 300mg
   □ Lanadelumab (Takhzyro) every 4 weeks 300mg
   □ Lanadelumab (Takhzyro) every ________ weeks 300mg
   □ Berinert every 2-3 days (dose depending on the body weight)
   □ Cinryze every 2-3 days (dose depending on the body weight)
   □ Orladeyo 150 mg once daily
   □ Others: ....

5. What was your main reason for taking LTP?
   (you can chose multiple answers!)

   □ High frequency of attacks
   □ Very painful attacks
   □ Dangerous attacks (for example angioedema of the throat)
   □ The number of absences from work/school due to HAE
   □ The burden of HAE in daily life
   □ The fear that an attack may occur at any time
   □ Others:...
   ....

6. Which angioedema trigger factors do you notice?

   □ Stress (in a positive and negative manner)
   □ Changes of temparature
   □ Hormonal factors (like menstruation)
   □ Mechanical stimuli (like injuries)
   □ Infection (like to have a cold)
   □ Others:...
   ....

7. Did you experience any attacks since initiating LTP?

➔ Please write down in detail:

**Date**	**Possible Trigger**<br><br>**(like infection)**	**Body region of edema**<br><br>**(like hand or abdominal pain)**	**Intensity of the attack (mild, moderate, heavy)**

8. Which medication do you take for acute treatment of an attack?

□ Icatibant 30 mg SC
□ Berinert IV, dose depending on the body weight
□ Others: ....
....

9. Did you undergo any kind of surgery since the initiation of LTP? If yes: what kind of surgery and when? If you underwent several operations, please list them all separately.
....
....
....
....

10. If you answered question 9 with „yes“: Did you take any prophylactic treatment for HAE as short-term-prophylaxis before surgery? If „yes“: which treatment? (for example Berinert IV). If you underwent several operations: please list them separately.
    ....
    ....
    ....
    ....

11. If you answered question 9 with „yes“: Did you suffer from angioedema after the surgery? If „yes“, please list the body region of edema, the intensity (mild, moderate or heavy) and the acute treatment. If you underwent several operations: please list the answers separately.
    ....
    ....
    ....
    ....

12. Did you undergo any kind of dental procedure since the initiation of LTP? If „yes“: what kind of dental procedure? (Please also list professional teeth cleaning) If you underwent several procedures, please list them all separately.
    ....
    ....
    ....
    ....

13. If you answered question 12 with „yes“: Did you take any prophylactic treatment for HAE as short-term-prophylaxis before surgery? If „yes“: which treatment? (for example Berinert IV). If you underwent several procedures: please list them separately.
    ....
    ....
    ....
    ....

14. If you answered question 12 with „yes": Did you suffer from angioedema after the dental procedure? If „yes", please list the body region of edema, the intensity (mild, moderate or heavy) and the acute treatment. If you underwent several procedures: please list the answers separately.
    ....
    ....
    ....
    ....

15. Did you undergo any kind of gastroscopy or colonoscopy since the initiation of LTP? If „yes": which procedure? If you underwent several procedures, please list them all separately.
    ....
    ....
    ....
    ....

16. If you answered question 15 with „yes": Did you take any prophylactic treatment for HAE as short-term-prophylaxis before? If „yes": which treatment?
    (for example Berinert IV). If you underwent several procedures: please list them separately.
    ....
    ....
    ....
    ....

17. If you answered question 15 with „yes": Did you suffer from angioedema after the procedure? If „yes", please list the body region of edema, the intensity (mild, moderate or heavy) and the acute treatment. If you underwent several procedures: please list the answers separately.
    ....
    ....
    ....
    ....

18. Did you experience any kind of trigger factor since the initiation of LTP? (like infection, illness, stress....)? If yes, please specify.
    ....
    ....
    ....
    ....

19. Please characterize the angioedema attacks that occured after the trigger factors of question 18. Please list the body region of edema, the intensity (mild, moderate or heavy) and the acute treatment.
    ....
    ....
    ....
    ....

20. Did you notice „prodromal symptoms" before an HAE attack **before** taking LTP? (like tiredness, changes of mood, nausea,...)? If yes, please list them all:
    ....
    ....
    ....
    ....

21. How did you react when you noticed those prodromal symptoms? (like „nothing/waiting", taking acute treatment for HAE, trying to relax...)?
    ....
    ....
    ....
    ....

22. Did you notice prodromal symptoms **after** the initiation of LTP? If „yes", what kind of prodroml symptoms? Please list all of them.
    ....
    ....
    ....
    ....

23. Did you suffer from angioedema attacks after the occurence of prodromal symptoms since taking LTP? Please list all of them.
....
....
....
....

24. Did those prodromal symptoms change since yout take LTP? (Like changes in frequency, intensity,...) If „yes", please specify.
....
....
....
....

25. How do you react when you notice those prodromal symptoms **since you take LTP**? (like „nothing/waiting", taking acute treatment for HAE, trying to relax...)?
....
....
....
....

26. Did you ever have a red exanthema of the skin before an angioedema attack? (so called „Erythema marginatum")
....
....
....
....

27. Did you ever have a red exanthema of the skin before an angioedema attack **since taking LTP**? (so called „Erythema marginatum")
....
....
....
....

28. Which are the major advantages of taking LTP for you?
....
....
....
....

29. Do you have any ideas, how to improve LTP? Is there anything that should be better?
....
....
....
....

Thank you very much!

Your team of the angioedema center Ulm University Hospital, ORL department
